# De-Politicizing Youth Suicide Prevention

**DOI:** 10.3389/fped.2013.00008

**Published:** 2013-05-02

**Authors:** Said Shahtahmasebi

**Affiliations:** ^1^Centre for Health and Social Practice, Wintec, HamiltonNew Zealand; ^2^The Good Life Research Centre TrustChristchurch, New Zealand; ^3^Voluntary Faculty, University of KentuckyLexington, KY, USA

**Keywords:** public health, mental health, community-based action

## Abstract

Despite a large volume of research literature on suicide, the approach to suicide prevention is still firmly based on a medical model. Recently, the Chief Coroner in New Zealand expressed the view that current techniques have failed to reduce the suicide rate and a new approach is needed. However, the call for a new approach is often interpreted as disparities in access to mental health services so resources are directed to increase public access to them. Current evidence suggests that persisting with depression and mental illness as a rationale for suicide prevention is unwise and is highly politicized. For example, over the last decade or so, despite a sustained awareness campaign on depression and mental illness and the doubling of prescriptions for anti-depressants, suicide rates maintained an increasing trend over the same period. It is argued that a new approach must redefine the suicide prevention problem holistically so that the whole community may share ownership of the problem. This paper argues that in order to move forward with a new approach, suicide prevention must be de-politicized – and describes a grassroots approach to de-politicization. Initial results suggest that with the grassroots approach there is potential to save lives, and it is cost-effective and sustainable.

## Introduction

New Zealand has one of the highest suicide rates in the OECD. Typically, suicide is often referred to as a major public health concern but in practice it is classed as a mental health issue for intervention and prevention policy development. The New Zealand approach to prevention is a one dimensional medical model with a moratorium on suicide reporting in the media which has led to a culture of secrecy. In an earlier attempt, the New Zealand Government’s suicide prevention strategy document (1) demonstrated a move away from the medical model by including all other possible factors reported in the literature as potential contributors: from alcohol and drug abuse to bereavement, family break-up, unemployment, educational and financial failure, and so on. Yet, policy actions are based on a long established view that mental illness (specifically depression) causes suicide. For example, in 2006 the New Zealand Government claimed “We know that up to 90% of suicides are *caused* by depression and that each year 500 New Zealanders are dying by suicide.” Therefore, despite its own strategy document that listed a large array of socio-economic and environmental risk factors the $6.4 million campaign was focused on reducing the impact of depression (2). The problem is that, the Government’s statement is misleading because current estimates suggest that between two-third and three-quarters of all suicides do not have a first contact with psychiatric services (3, 4). And of those who do have a psychiatric record only a fraction have depression recorded as a diagnosis (4). Therefore the question arises how do we know that 90% of suicides are caused by depression?

As a consequence the guidelines for suicide prevention recommend that the public look for signs of mental illness and depression and refer the case to a mental health unit. But, waiting for symptoms to show up is not prevention. If symptoms are detected then an event has occurred, in which case it is time to intervene. Intervention is always difficult and unsustainable as a prevention strategy when there is little understanding of the nature of the problem.

The problem with such a prevention system is that it ignores the majority who do not exhibit symptoms or are good at hiding them, or who do not have them. In addition, the emphasis on mental illness as the main cause of suicide will make sure that suicide is treated as depression rather than “suicide” and reinforces its taboo status. As a result of the relentless emphasis on mental illness and depression as causes of suicide, prevention policies do not address suicide nor do they prevent suicide or depression occurring.

Clearly, a single-dimensional mind-set about suicide prevention means a highly politicized suicide prevention process, with grave implications for suicide prevention, research, and distribution of funds and resources. In this paper, I present a grassroots approach to the de-politicization of suicide prevention policy development.

## Materials and Methods

### Background: Processes and actors

**Figure 1:**
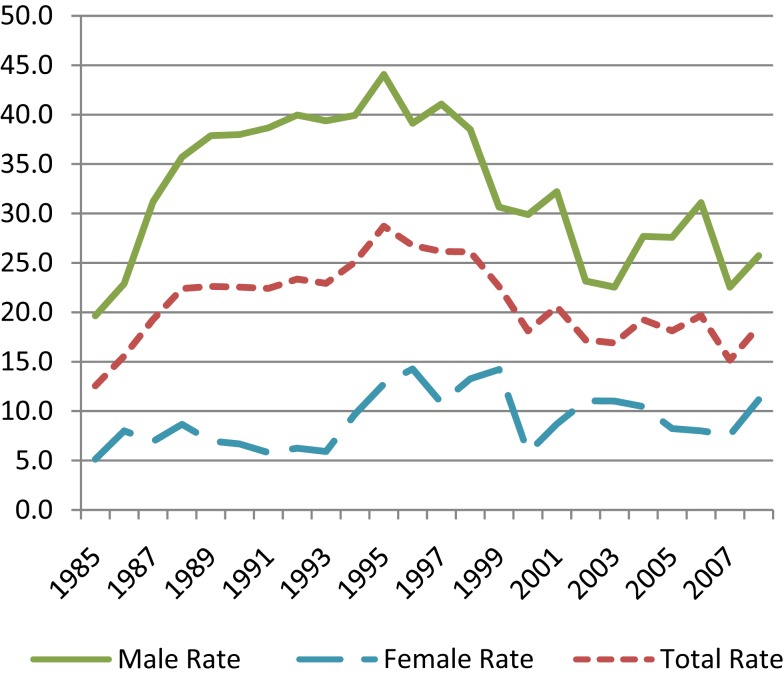
**Age-specific youth suicide rates by sex in New Zealand 1985–2008**. Source: Ministry of Health New Zealand Health.

**Figure 2:**
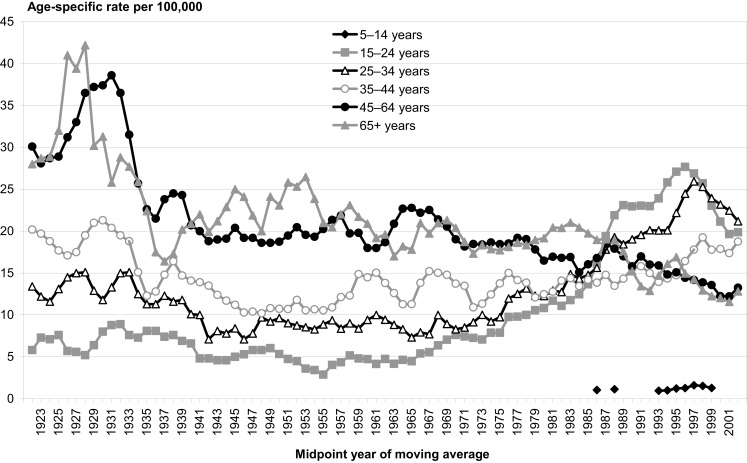
**Suicide rates by age, New Zealand 1923–2003**. Source: New Zealand Health Information Service.

A quick visual analysis of conflicting trends for youth suicide in New Zealand, Figure 1, suggests the presence of a cyclic effect as well as a lagging effect in male and female suicide trends. On average, a low point on the trend for females appears to coincide with a high point for the males at the same time point. This pattern seems to be repeated every 7–10 years approximately. A similar pattern can be observed for all suicides by age group, as in Figure 2. That is, while one group’s trend is decreasing another is increasing which can be observed in terms of the gender differences in Figure 1, and age-group differences in Figure 2.

This information should be informing the process of suicide prevention policy development. Yet, when the overall trend is slowing down the authorities claim that their policies are working. They then request more funding to apply the same policies to sub-group(s) whose trend is lagging or has completed the cycle and is increasing. And when the overall trend is upward the authorities claim that suicide is a very complex issue with many risk factors including socio-economic, environmental, and mental illness factors, and request more funding to increase access to mental health services in particular for low income groups and young people.

This is fine the first time, but when it happens year after year and cycle after cycle, then suicide prevention becomes more of the same old interventions but at a higher costs in terms of lives lost and resources. Furthermore if mental illness and depression were direct causes of suicide then surely after decades of treating suicide for depression we should have observed a decreasing trend [also see Ref. (5)] rather than continuing cycles. Moreover, between 1997 and 2005 prescriptions for anti-depressants had doubled in New Zealand (6) and since then it has doubled again (7), whilst over the same period the suicide rate has increased. The findings, collected by the Ministry of Justice, show suicide in New Zealand has risen from 540 deaths annually in 2007/2008 to 558 in 2010/2011 (8). It is not surprising that in 2011 the New Zealand chief coroner stated that current methods are not working and called for a new approach to suicide prevention (8).

The government’s own policy documents list a large number of risk factors, it is interesting that these are often translated into policy actions for mental illness intervention (9). A single model for suicide prevention based on unproven cause and effect will be limited to the politics of a top-down approach, i.e., “experts” vs. the public, and, political gestures. For example, in 2011 the Associate Health Minister chaired a meeting with media, mental health professionals, and researchers to update the guidelines on suicide prevention (10). There was no mention of involvement of communities or victims/survivors of suicide (family of a suicide case). Following public protests a spokesman for the Associate Health Minister suggested that suicide survivors would be able to participate in the meetings later in the year. Involving the public after decisions are made is merely a political gesture of no value.

Associated with political gestures is the release of confirmed suicide data. For example, in August 15, 2012, the Associate Minister for Health stated (11) “A total of 522 people died by suicide in New Zealand in 2010, or 11.5 deaths per 100,000 people. As a proportion of the population, this is 23.6 per cent below the peak of 577 in 1998, but up slightly on the 510 deaths in 2009.” Eleven days later on August 26, 2012, the chief Coroner stated that suicide in New Zealand had risen from 540 deaths annually in 2007/2008 to 558 in 2010/2011 (8). Clearly, the chief coroner’s statement about New Zealand’s suicide trends describes a scenario worse than that described by the Minister for Health.

Over two-thirds of cases do not come into contact with mental health services. Various attempts to include these cases in research have made such studies highly biased because of design and analytical methodologies that fail to account for sources of bias. First, researchers and authorities have established depression and mental illness in the public mind-set as causes of suicide. Second, these same researchers collect statements about the suicide cases’ mental wellbeing from family and friends after the event of suicide. It is no wonder that time and again this type of research leads to erroneous results and mis-conclusions that mental illness is the major cause of suicide (12). The flipside of the coin is the negative and undesired consequences of policy based on erroneous results, e.g., increased antidepressant prescriptions [including very young children (12)].

The main actors in the current suicide prevention system are the government who controls the resources, and the “expert” advisors. Various organizations, including mental health units, who have modeled their care services on the government suicide prevention guidelines, compete for resources. Naturally, politics is a main feature in policy development leading to top-down policy actions, and excludes discussion of alternative approaches that do not totally overlap with the current model of suicide prevention. Change and flexibility in the model are overdue.

### Conceptualizing a grassroots approach

In order to address a problematic issue, the nature of the problem must be understood. The fact is, at the center of each suicide there is a human being with his/her family and a social community network. We may not understand suicide but there is capability in the literature to address aspects of human behavior. However, the large number of variables reported in the literature as risk factors suggests that the public at large is at risk of suicide. In other words, everyone can potentially be exposed to life changing events and therefore at risk of suicide, e.g., divorce, illness (physical or mental), unhappiness, too much happiness, employment issues, financial difficulties, loss and bereavement, relationship issues. Thus, the main actors in a suicide prevention strategy must be the public. To attenuate the link between suicide prevention policy and politics the main actors must take ownership of the suicide prevention problem.

**Figure 3:**
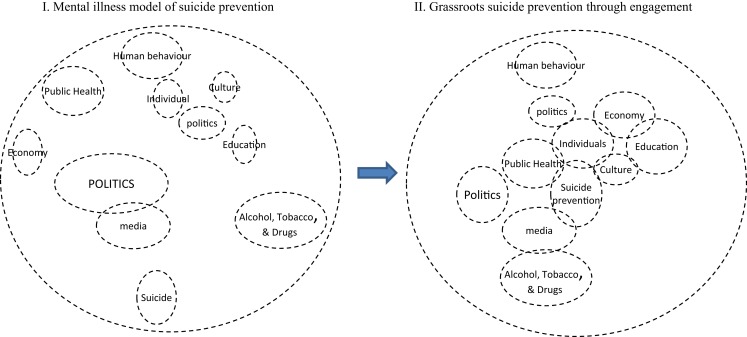
**Graphic visualization of suicide prevention**.

To conceptualize a dynamic model for collaboration suicide must be placed at the center of this model, see Ref. (13), and merge current knowledge while seeking new information and updating our understanding of suicide, Figure 3. In order to emphasize positive suicide prevention, the influence from all relevant processes (including the negative effects from erroneous policies) must be equalized. With such a conceptualization, de-politicization is a natural process due to the willingness to collaborate rather than one discipline dominating others.

It can be visualized that all processes are interconnected (Figure 3) through temporal dependencies of all aspects of life and the subsequent feedback effect. Suicide prevention must follow a holistic grassroots approach to allow for complexities due to environmental, social, and health processes. It is important to anticipate the feedback effect from each policy decision within these processes to prevent policy and policy makers becoming part of the problem (13).

A subsequent and natural step of the conceptualization process (13) was to engage the main actors, i.e., members of the public. This idea utilizes the local community/public and local knowledge to address local issues, e.g., see Refs. (14–16). In order for a community approach to suicide prevention to be relevant and appropriate some insight into the community’s understanding and perceptions of suicide and suicide prevention was necessary. This issue was easily addressed by linking in with communities at a local level, focusing on adolescent health and youth suicide prevention and securing the commitment of an international adolescent health expert (16) to contribute to the project. Raising funds was not so straightforward despite a sympathetic Associate Minister of Health (in 2006) and international support for a new approach.

A key component of the conceptualization was to engage community agencies in working together for a common goal. Community agencies brought together, supported by a tertiary education provider, to operationalize the suicide prevention at grassroots project in 2009/2010.

### Operationalizing a grassroots approach

For the approach to be successful it had to address the needs of the participating communities as perceived by them. In order to address this problem informal information gathering was conducted. Frontline health workers from three communities in Waikato, New Zealand, were contacted for their perceptions of the community’s needs in the context of suicide prevention. The frontline health workers indicated that their greatest need was for information, training, and for upskilling in order to be able to deal with youth and adolescent issues. The resulting outcome was a pilot project offering training workshops.

The frontline health workers organized the community workshops including venues, publicity, invited local dignitaries, and other community members, e.g., police, teachers, social workers, counselors, young people, and the general public. The project intended to empower communities to plan and make decisions at family and community level by increasing their awareness of adolescence issues. In this context the role of the researchers was to facilitate training workshops and basically play a support and mentoring role. All the community projects and activities that followed were designed and developed at grassroots level by the communities themselves.

## Results

The 2010 pilot workshops identified a number of important issues such as public frustration with the secrecy surrounding the suicide debate, a lack of preparedness of public and health workers to intervene early, a lack of appropriate support for suicide survivors, and a great need for training (17, 18). The public demand and requests for repeat workshops provided the evidence needed for community training in suicide prevention and a follow-up was organized in 2011. The 2011 workshops were funded by: a Fulbright specialist grant, Waikato Institute of Technology (Wintec), participating communities, and Trust Waikato (a local Charity).

Once again, the workshops were organized by community liaisons who were the frontline health workers representing their communities. The workshops were presented by an international “adolescent medicine” expert (16). Based on feedback from the first workshop series, the follow-up workshops were developed to cover the knowledge and skills gap in each community. The training materials were designed to tackle suicide prevention more holistically by understanding adolescence and adolescent behavior. The key message of these programs is that suicide is not a solution to problems, and that the community cares (16).

Attendees at the workshops included health workers, community police, educators, students, counselors, suicide survivors (families of suicide cases), and the general public. The main issue that was identified during the pilot project and follow-up workshops was the secrecy around suicide which has led to public silence. Suicide survivors want to be able to talk about their experiences and to contribute to suicide prevention but they felt that no one was listening. The frontline workers felt they were unprepared for suicide prevention, and, that intervention was restricted to following the official guidelines of looking for signs of depression and then referring to mental health services.

**Figure 4:**
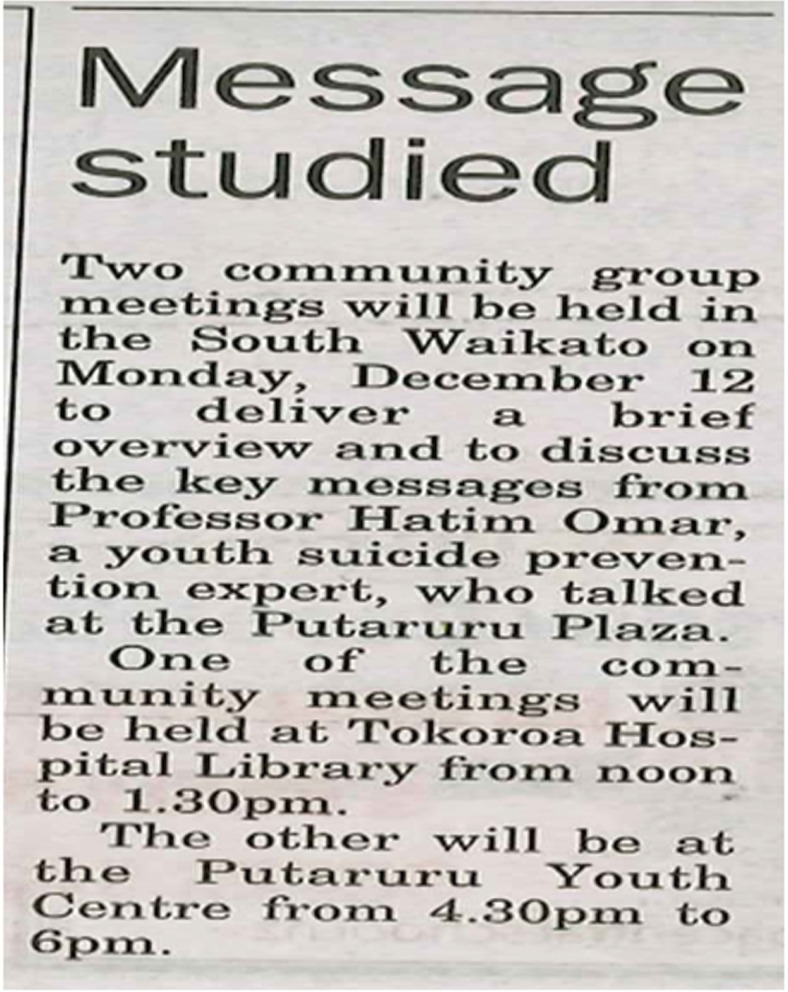
**Local media announcement about suicide prevention groups**.

The evaluations for the workshops were 100% positive and armed with additional and new knowledge, communities set about devising plans to prevent or intervene in suicide, see Figure 4. Establishing a local suicide prevention group to help with planning and operational issues followed by more talking were the key actions decided by the groups. Some communities worked faster than others and developed more ideas, for example: one group organized suicide awareness activities (19), e.g., suicide awareness street festival that included quizzes, t-shirts, surveys with prizes, leaflets about the availability of and how to contact community and medical support, “shout-out” cards where the cardholder is encouraged to list people they would contact for help or to talk to. Interestingly, a local newspaper has been running regular articles on suicide (19), and some communities reported lives saved as a result.

The grassroots project aspired to inform communities so that they make informed choices about suicide prevention as opposed to telling them what to do, how to do it, and not to talk about it. As a result, the communities were empowered to mobilize themselves to address suicide, see Figure 4. One of the main achievements of such suicide prevention groups was to engage the whole community including local businesses, clubs and societies, health services, whether by contributing funds, sponsorship, free advice, or resources (e.g., donating materials, manpower, venues).

## Discussion and Conclusion

It is interesting to note that the actions and activities that the communities had organized are all designed to engage the community at grassroots including open discussion and debate. As a result, actions at grassroots refocus suicide and address social, community, and individual parameters, such as “the community cares,” or “talking and listening to your children,” “listening and talking to your neighbor/friend,” these actions will tackle the many risk factors reported in the literature, e.g., loss, divorce, and reverse the perception that suicide is a valid option. This approach also highlights the availability and importance of interventional mental services.

Through engaging the community (e.g., simply talking and the use of shout-out cards) at least two potential suicides have been prevented. This suggests that the secrecy and taboo status of suicide must be lifted in order for communities to be empowered to care for themselves. Grassroots-level action does not label people with mental illness categories or alienate them. Over time, it is more likely that individuals will talk about their issues and seek appropriate help rather than suffer and make life and death choices in isolation and silence.

The most striking impression with the grassroots approach is how quickly the community mobilized itself (within 6–7 months of the follow-up workshops) to own the problem and respond, with very little resources and no funding. Since the workshops, there has been a drop in youth suicide in the two communities that adopted the grassroots approach to suicide prevention. At the time of writing, the number of youth suicides in South Waikato had reduced considerably: from an average of one youth suicide per month up to February 2012 (2 months after the workshops) to two in the period February–November. However, one of the cases had traveled from another area, and, over the same period there was one adult suicide who was receiving treatment from mental health services.

Whether or not the grassroots approach will work to reduce suicide rates within participating communities is under investigation. However, it must be noted that empowering and mobilizing the community to respond to a problem at community and local level, was very quickly organized, inexpensive, and effective both the short-term and long-term, i.e., it is a sustainable policy. It is effective because the process of decision making, albeit with a focus on reducing suicide, considers local processes, such as education, social, and economic environment.

## Conflict of Interest Statement

The authors declare that the research was conducted in the absence of any commercial or financial relationships that could be construed as a potential conflict of interest.
